# The complete chloroplast genome of *Callicarpa rubella* Lindl. (Lamiaceae) from Guangxi, China: genome structure and phylogenetic analysis

**DOI:** 10.1080/23802359.2021.1993106

**Published:** 2021-10-23

**Authors:** Huimin Cai, Xing Liu, Zhiwei Su, Zhonghui Ma

**Affiliations:** a National Demonstration Center for Experimental Plant Science Education, Traditional Chinese Herbal Medicine Resources and Agriculturalization Research Institute, College of Agriculture, Guangxi University, Nanning, China; b Institute of Marine Drugs, Guangxi University of Chinese Medicine, Nanning, China

**Keywords:** *Callicarpa rubella*, chloroplast genome, Lamiaceae, phylogenetic relationship

## Abstract

The relationship between *Callicarpa rubella* Lindl. and its infraspecific taxa has troubled researchers for a long time. Here, we reported for the first time the complete chloroplast (cp) genome of *C. rubella* to investigate its phylogenetic position and provide more sequencing information for further studies about the relationship between *C. rubella* and its related species. The cp genome of *C. rubella* was 154,202 bp in length and displayed a typical quadripartite angiosperm structure, containing two inverted repeat (IR) regions of 25,701 bp, a large single-copy (LSC) region of 84,968 bp and a small single-copy (SSC) region of 17,832 bp. It contained 87 protein-coding genes, 37 tRNA genes and 8 rRNA genes. The analysis fully resolved *C. rubella* was in a clade with *C. bodinieri* and *C. nudiflora*. The results indicated *Callicarpa* formed a sister relationship with *Dicrastylis parvifolia* in Lamiaceae.

*Callicarpa* (Lamiaceae) with the nickname ‘beauty berry’ was first described by Linnaeus (1753) and due to its attractive purple fruits usually displaying in the autumn, the genus takes the name. The genus as traditionally medicinal plant group harbors significantly economic and pharmacological importances (Tu et al. 2013). *Callicarpa* was traditionally assigned to Verbenaceae, however subsequently Harley et al. (2004) adopted transferring it to Lamiaceae. *Callicarpa rubella* Lindl. 1825 is a shrub, distributed widely in eastern and southeastern Asia (China, Indonesia, Malaysia, Myanmar, Thailand and Vietnam) and always grows in some easily disturbed areas (roadsides or the margin of secondary forest). *C. rubella* is variable morphologically, especially in its indumentum, and the size and shape of the leaves. It is always a headache to identify *C. rubella* from its infraspecific taxa (*C*. *rubella* var. *subglabra*, *C. rubella* f. *angustata* and *C. rubella* f. *crenata*) and some related species (*C*. *longipes* and *C*. *mollis*) based on morphologies. In this study, we assembled and annotated the complete chloroplast (cp) genome of *C. rubella* for the first time to serve as a genetic resource for future studies on the taxonomy of *Callicarpa* and to get a better understanding of phylogenetic relationships in this pantropical genus.

The fresh leaves of *C. rubella* were collected from Guiping, Guangxi, China (23°41’10′’N, 110°1’12′’E). A specimen was deposited at the herbaria in College of Agriculture, Guangxi University (https://nxy.gxu.edu.cn/, Zhonghui Ma, mazhonghui@gxu.edu.cn) under the voucher number H004. The total genomic DNA was extracted by modified CTAB method (Doyle and Doyle 1987) and used for sequencing on Illumina NovaSeq 6000 platform at the Beijing Novogene Technology Co., Ltd. (Tianjin, China). The DNA sample was deposited in the Plant Systematic Evolution Laboratory, College of Agriculture, Guangxi University. The cp genome was assembled by GetOrganelle toolkit (Bankevich et al. 2012; Langmead and Salzberg 2012; Wick et al. 2015; Jin et al. 2020) and annotated using Plastid Genome Annotator (PGA) (Qu et al. 2019). Aligning the complete cp genome and manually adjusting annotation were performed in Geneious (Kearse et al. 2012). The annotated cp genome has been deposited in GenBank (accession number: MZ520129).

The cp genome of *C. rubella* was 154,202 bp in length and had a typical quadripartite angiosperm structure, containing two inverted repeats (IRA and IRB), each of 25,701 bp, a large single-copy (LSC) region of 84,968 bp and a small single-copy (SSC) region of 17,832 bp. The GC content of the whole cp genome was 38.1%. We recovered a total of 128 distinct genes, including 87 protein-coding genes (PCGs), 37 tRNA genes and 8 rRNA genes. Of these genes, 18 were duplicated, including 7 protein-coding, 7 tRNA and 4 rRNA genes.

To confirm the phylogenetic position of *C. rubella* with related species, 24 chloroplast (cp) genomes sequences were downloaded from GenBank, including 22 species from Lamiaceae representing 12 subfamilies recognized currently and 2 outgroup species from Mazaceae and Phrymaceae (Figure. 1) (Vallejo-Marin et al. 2016; Ha et al. 2018; Tao et al. 2019; Wang et al. 2019; Du et al. 2020; Zhao et al. 2020; Wang et al. 2021; Xie et al. 2021; Zhao, Chen, et al. 2021; Zhao, Wu, et al. 2021). Due to the rapid development of molecular phylogenic studies during the past two decades, the traditionally systematic positions of some genera have dramatically changed. *Congea*, *Vitex* and *Tectona* (originally belong to Verbenaceae) have been moved to Lamiaceae (Harley et al. 2004) and *Mazus* and *Erythranthe* (formerly Scrophulariaceae) have been assigned to Mazaceae (Reveal 2011) and Phrymaceae (Beardsley and Olmstead 2002; Tank et al. 2006) separately, recognized by APG IV (Angiosperm Phylogeny Group 2016). The sequences were aligned with MAFFT (Katoh and Standley 2013) performed in Geneious and the phylogenetic relationship was conducted using RAxML-HPC2 on XSEDE at CIPRES Science Gateway (Miller et al. 2010) with the GTR Gamma and 1000 bootstrap replicates based on a data matrix of concatenation of 78 coding protein sequences (CDS). The analysis fully resolved *C. rubella* was in a clade with *C. bodinieri* and *C. nudiflora* (Figure 1). The results indicated *C. rubella* and other species of *Callicarpa* located at the bottom of the phylogenetic tree in Lamiaceae and formed sister relationship with *Dicrastylis parvifolia* which verified the previous conclusions about the original position of *Callicarpa* in the family Lamiaceae (Bramley et al. 2009; Drew and Sytsma 2012; Li et al. 2016) (Figure 1). This study also provided important sequence information for the coming solution about the relationship between *C. rubella* and its related species.

**Figure 1.**
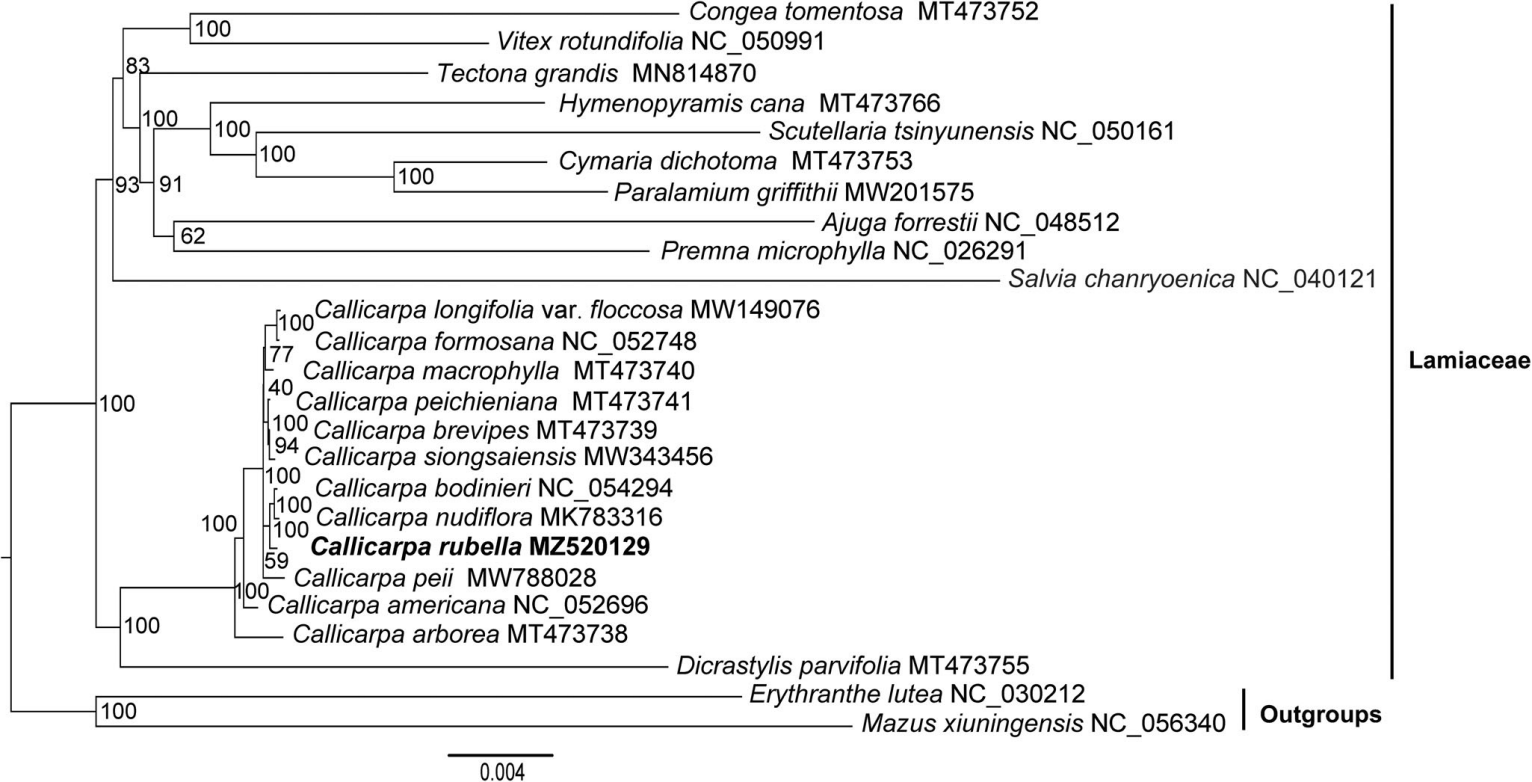
The maximum-likelihood (ML) phylogenetic tree of 25 taxa based on 78 CDS of the chloroplast genomes. Numbers near the branches are bootstrap support values. The *C. rubella* genome was marked in bold font.

## Data avaliability statement

The complete chloroplast genome data that supports the findings of this study are openly available in GenBank of NCBI at https://www.ncbi.nlm.nih.gov/ under the accession no. MZ520129. The associated BioProject, BioSample and SRA numbers are PRJNA744154, SAMN20080096 and SRR15049219, respectively.
